# Infiltrative microgliosis: activation and long-distance migration of subependymal microglia following periventricular insults

**DOI:** 10.1186/1742-2094-2-5

**Published:** 2005-01-28

**Authors:** W Shawn Carbonell, Shin-Ichi Murase, Alan F Horwitz, James W Mandell

**Affiliations:** 1 Medical Scientist Training Program, University of Virginia, Charlottesville, Virginia 22908, USA; 2 Neuroscience Graduate Program, University of Virginia, Charlottesville, Virginia 22908, USA; 3 Department of Cell Biology, University of Virginia, Charlottesville, Virginia 22908, USA; 4 Department of Pathology (Division of Neuropathology), University of Virginia, Charlottesville, Virginia 22908, USA

## Abstract

**Background:**

Subventricular microglia (SVMs) are positioned at the interface of the cerebrospinal fluid and brain parenchyma and may play a role in periventricular inflammatory reactions. However, SVMs have not been previously investigated in detail due to the lack of a specific methodology for their study exclusive of deeper parenchymal microglia.

**Methods:**

We have developed and characterized a novel model for the investigation of subventricular microglial reactions in mice using intracerebroventricular (ICV) injection of high-dose rhodamine dyes. Dynamic studies using timelapse confocal microscopy *in situ *complemented the histopathological analysis.

**Results:**

We demonstrate that high-dose ICV rhodamine dye injection resulted in selective uptake by the ependyma and ependymal death within hours. Phagocytosis of ependymal debris by activated SVMs was evident by 1d as demonstrated by the appearance of rhodamine-positive SVMs. In the absence of further manipulation, labelled SVMs remained in the subventricular space. However, these cells exhibited the ability to migrate several hundred microns into the parenchyma towards a deafferentation injury of the hippocampus. This "infiltrative microgliosis" was verified *in situ *using timelapse confocal microscopy. Finally, supporting the disease relevance of this event, the triad of ependymal cell death, SVM activation, and infiltrative microgliosis was recapitulated by a single ICV injection of HIV-1 tat protein.

**Conclusions:**

Subependymal microglia exhibit robust activation and migration in periventricular inflammatory responses. Further study of this population of microglia may provide insight into neurological diseases with tendencies to involve the ventricular system and periventricular tissues.

## Background

It has become increasingly evident that the central nervous system is an immunocompetent organ [1]. Microglia are the primary immune effector cells of the brain parenchyma and functionally resemble tissue macrophages elsewhere in the body [1,2]. The brain ventricles are also under immune surveillance by intraventricular macrophages which patrol the cerebrospinal fluid (CSF), choroid plexus, and supraependymal surface [3]. At the interface of the CSF and brain proper is the ciliated neuroepithelial ependymal cell which lines the ventricular system of the brain and spinal canal. The ependyma not only functions as a physical barrier preventing foreign proteins and organisms from entering the brain from the CSF, but also displays immunological effector ability such as phagocytosis of fluorescent beads injected into the CSF [4] and upregulation of MHC-II in response to interferon gamma challenge *in vivo *[5]. These diverse cell types may work in concert establishing the basis for the innate immune system of the CNS.

Importantly, a population of resident subventricular microglia (SVMs) are found in the subependymal zone [6,7] suggesting the ependyma and microglia may cooperate to prevent invasion of the CNS from the ventricular system. Juxtaventricular microglia have been shown to react to both direct periventricular/ependymal damage as well as the mere presence of cytokines in the CSF. For instance, the intracerebroventricular (ICV) injection of lentiviral tat protein in low nanomolar quantities is sufficient to kill ependymal cells and cause a periventricular inflammatory reaction including the characteristic microglial nodules of human HIV-1 encephalopathy (HIVE) [8]. Alternatively, Kong et al [9], noting the high CSF levels of inflammatory cytokines in multiple sclerosis (MS), demonstrated a vigorous periventricular activation of microglia with ICV injection of IFN-γ alone or in combination with endotoxin or TNF-α. In these cases, microglia were activated in the absence of primary tissue damage, but were thought to contribute secondarily to immune-mediated periventricular damage via potentiation of cytokine release and bystander effect. Of note, both HIVE and MS often present with enigmatic periventricular inflammatory lesions in humans [10-13].

The reaction of SVMs to periventricular damage has not been described in detail. Specifically, the functional repertoire displayed by activated SVMs including phagocytosis and long-distance migration have not been investigated. Here, we directly examine SVM function by exploiting a novel methodology which selectively activates and labels SVMs *in vivo *combined with confocal timelapse techniques for dynamic analyses in adult mouse brain tissue. We hypothesized that SVM activation is a general consequence of periventricular insults that can be caused by diverse circulating substances in the CSF known to damage the ependyma. Our work indicates that activated, phagocytic SVMs are capable of infiltrating deep within the parenchyma.

## Methods

### Animals

Adult male C57bl/6 mice (6–8 wks) were obtained commercially from Harlan (Indianapolis, IN) and cared for in accordance with Public Health Service and University of Virginia guidelines.

### Surgical procedures

To damage the ependyma 2–3 μl of 0.2% Sp-DiI (D-7777, Molecular Probes) in DMSO; 1:20 rhodamine-conjugated latex microspheres (Lumafluor, Naples, FL) in sterile PBS; 0.25 U Neuraminidase (Sigma); or 2.0 nM recombinant HIV-1 tat protein in 100 mg/ml BSA, 0.1 mM DTT in PBS were stereotaxically injected into the left lateral ventricle at the following coordinates: L, 1.5 mm; P, -0.5 mm; D, 2.0 mm. GFP-expressing adenovirus (10^9 ^plaque forming units); 100 mg/ml BSA, 0.1 mM DTT in PBS, or deactivated tat [14] were used as volume-matched controls. Forebrain stab lesion for deafferenting lesion of the contralateral hippocampus was performed as previously described [15]. Briefly, mice were anesthetized with a Xylazine/Ketamine mixture and placed in a stereotaxic head holder (Benchmark, myneurolab.com). Temperature was maintained with a ventral heat pad. A right parietal craniectomy extending 3–4 mm from midline and spanning Lambda to Bregma was created with a microdrill. Beginning at the level of Bregma, a 3 mm lesion was created in the sagittal plane 1.5 mm from midline at a depth of 3.5 mm with a sterile no. 11 scalpel blade held in the stereotaxic device. After achieving hemostasis, the bone was replaced and sealed with carboxylate cement (Durelon, Fisher Sci). This right sided lesion results in deafferentation injury to primarily *stratum oriens *of the left hippocampus.

### Static analyses

Brains were collected and processed for histopathology as previously described [15]. All antibodies and staining procedures have been described previously [15] except for rat anti-cd11b/MAC-1 (1:50), mouse anti-foxj1 (1:1000), mouse anti-nestin/rat-401 (1:100), and rat anti-F4/80 (1:10). Histochemistry for biotinylated *Griffonia simplicifolia *lectin IB_4 _was performed 1:100 in PBS overnight at 4°C and visualized with either 1:200 FITC- or Alexa Fluor 350-conjugated streptavidin (Sigma and Molecular Probes, respectively).

### Dynamic analyses

200–400 μm live slices were prepared from adult C57BL/6 mice as described previously [16,17]. Briefly, mice were acutely anesthetized in a chamber with halothane and decapitated. The brain was rapidly removed, blocked, and covered with 10% agar at 37°C in a specimen mold (Tissue-Tek 4566, Fisher). Live slices were obtained with a vibratome and placed individually on Millicell-CM inserts (Millipore PICM03050, Fisher). Culture medium consisted of CCM1 (Hyclone, Logan, UT) with 20% heat-inactivated normal horse serum. Vital labelling of microglia in tat experiments was performed with Alexa 488 or 568 IB_4 _(Molecular Probes) [17]. Laser-scanning confocal images were acquired on a Nikon IX-70 inverted microscope with Fluoview 300 software (Olympus). A z-series stack covering 40 μm of slice thickness was taken every 1.5–4 minutes, creating a three-dimensional timelapse data set. To create timelapse movies from the data set, 4 to 6 z-plane images were collapsed as 2D projections using ImageJ 1.31 u and compiled into quicktime movies with Quicktime Pro 6.3. Movies were analyzed for migration speed and distance as described [18]. Values represent the mean ± SEM. Statistical analysis was performed with ANOVA or student's *t*-test. Pairwise post-hoc analysis was performed with a *t*-test and the Bonferroni correction factor. A *p *< 0.05 was considered statistically significant. Rose plots were created in Kaleidagraph 3.0.

## Results

### Selective ependymal death is induced by high-dose rhodamine dyes

**Figure 1:**
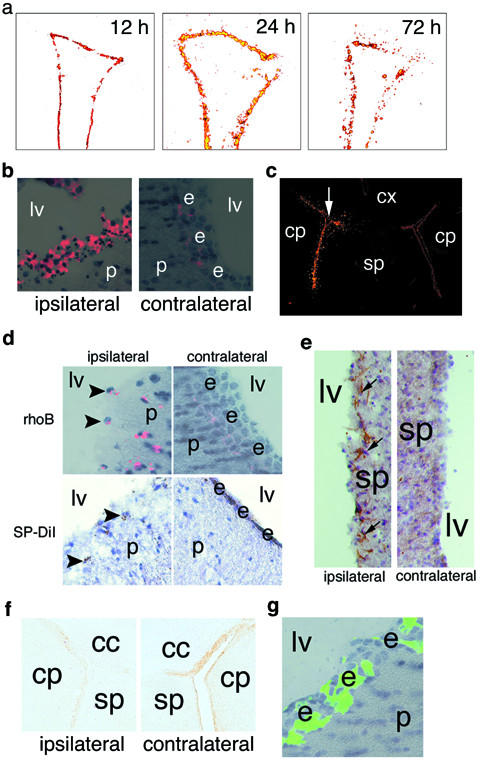
Ependymal damage with rhodamine dyes. (A) Timecourse of ependymal death in the lateral ventricle after rhodamine dye injection demonstrated with digital subtraction. Damage to the ependyma was evident at 12 h and rapidly progressed by 24 h. (B) Histology at 24 h demonstrates swollen ependyma with numerous pyknotic profiles in injected, but not the contralateral, hemisphere. e, ependyma; lv, lateral ventricle; p, parenchyma. RHO fluorescence overlaid on brightfield hematoxylin images. (C) Low-power view of lateral ventricles 3 d after injection demonstrates halo of rhodamine-positive cells around injected ventricle (white arrow). The contralateral ventricle demonstrates labelled ependyma in the absence of damage. (D) By 3 d, near-complete loss of the ependyma was evident. This coincided with the appearance of dye-laden SVMs, black arrowheads. The ependyma remained intact in the contralateral hemisphere (right panels). e, ependyma; lv, lateral ventricle; p, parenchyma; RhoB, rhodamine beads. RHO fluorescence overlaid on brightfield hematoxylin image (RhoB) and photoconverted DiI counterstained with hematoxylin. (E) Periventricular reactive astrocytes (black arrows) visualized with nestin immunohistochemistry (IHC) at 3d post-injection at wall of injected ventricle (left), but not in the contralateral hemisphere (right). lv, lateral ventricle; sp, septum. (F) IHC for ciliated cell-specific foxj1 28d after dye injection demonstrates persistent loss of ependyma in injected hemispere (left). cc, corpus callosum, cp, caudate/putamen; sp, septum. (G) Equivalent volume control injection of GFP-reporter adenovirus demonstrates no ependymal damage 3 weeks after injection. e, ependyma; lv, lateral ventricle; p, parenchyma. GFP fluorescence overlaid on brightfield hematoxylin image.

Rhodamine dyes such as SP-DiI and rhodamine latex microbeads (RhoB) have been classically used for tract tracing studies *in vivo *and in fixed tissues [19,20]. Recently, intracerebroventricular (ICV) injection of these dyes into the CSF has also been shown to selectively label ependymal cells [4,21]. In pilot studies for other projects, we discovered doses of these dyes that, in addition to labelling, result in selective death and denudation of the ependyma (not shown). To investigate the timecourse of ependymal damage in response to rhodamine dyes we injected a toxic bolus of SP-DiI (0.2% in DMSO) or rhodamine latex microbeads (1:20 in PBS) into the left lateral ventricle of mice. Overt ependymal damage was appreciated beginning by 12 h (Fig. 1a, left panel) post-injection and progressed rapidly by 24 h (Fig. 1a, middle panel) where the ependyma appeared swollen and ragged with frequent pyknotic profiles (Fig. 1b). At these doses, damage was largely restricted to the lateral ventricle ipsilateral to the injection and the third ventricle (not shown) while sparing the contralateral lateral ventricle (Fig. 1b, c) suggesting a dose- and diffusion-dependent toxicity. Near complete ependymal cell loss occured in regions of the lateral ventricle proximal to the injection site within 3 days with both dyes (Fig. 1A, right panel &1D). Mild subependymal astrogliosis as revealed by nestin immunohistochemistry was also evident by 3d (Fig. 1E). The loss of ependyma was further confirmed by chronic loss of immunoreactivity for the ciliated cell-specific transcription factor foxj1 at 1 month after injection (Fig. 1f). Animals injected with equal volumes of GFP-reporter adenovirus (Fig. 1g) or low-dose rhodamine microbeads in PBS (1:50, not shown) demonstrated no ependymal loss or activation of subventricular microglia (SVMs, not shown). Thus, rhodamine dyes rapidly and selectively damage ependymal cells at high doses.

### SVMs phagocytose ependymal debris

**Figure 2:**
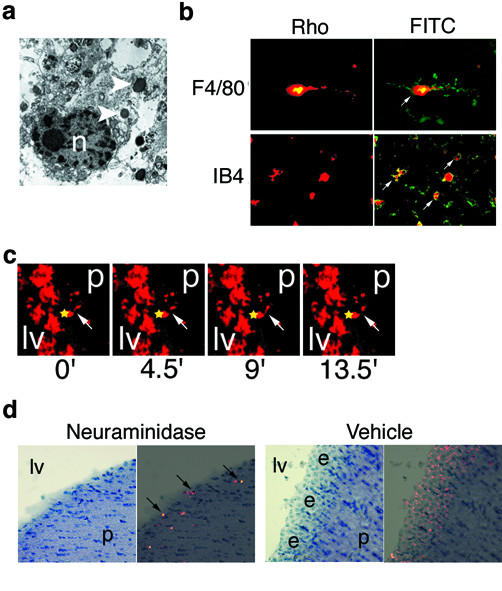
Selective labelling of SVMs with rhodamine dyes. (A) RHO+ cells are microglia. Transmission electron microscopy demonstrates dye-laden inclusions (white arrows) in a SVM. n, nucleus. (B) Immunohistochemistry for F4/80 (top) and histochemistry for lectin IB_4 _(bottom) demonstrate double-labelled periventricular cells, white arrows. (C) Time-lapse confocal microscopy in live brain slices demonstrates SVM (white arrow) extending (time 0' and 9') and retracting (time 4.5' and 13.5') a process toward ependymal debris (yellow star) highly suggestive of phagocytosis. See also Video 1. lv, lateral ventricle; p, parenchyma. (D) Neuraminidase injection following sublethal ependymal labelling similarly results in RHO+ SVMs (black arrows). e, ependyma; lv, lateral ventricle; p, parenchyma. Left panels, hematoxylin; Right panels, RHO fluorescence overlaid on hematoxylin.

Loss of the ependyma in the above areas coincided with the appearance of dye-laden periventricular cells resembling macrophages (Fig. 1d, arrowheads). At low power, affected ventricles were surrounded by a halo of these rhodamine-positive (RHO+) cells (Fig. 1a, last panel; 1c). To determine the identity of the RHO+ cells we performed transmission electron microscopy (Fig. 2a), immunohistochemistry for microglial/macrophage markers F4/80 (Fig. 2b) and MAC-1/cd11b (not shown), and histochemistry for IB_4 _lectin from *Griffonia simplicifolia *(Fig. 2b). These techniques demonstrated periventricular RHO+ cells to be microglia.

SVMs may become RHO+ as a result of phagocytosis of the dye-labeled ependymal debris. To provide direct evidence of this hypothesis we performed confocal time-lapse microscopy in living slices from adult mice given dye injection 24 h prior to sacrifice. Grossly, we observed a dramatic increase in RHO+ periventricular cells over several hours suggesting active clearance of labelled debris (not shown). Further, SVMs displayed dynamic behavior consistent with phagocytosis of ependymal debris (Fig. 2c, Video 1(Additional file 1)). Therefore, SVMs became rhodamine positive after high-dose dye injection due to phagocytosis of labelled ependymal debris.

To determine if SVM activation is a general response to periventricular damage we injected animals with a sublethal dose of RhoB to label ependymal cells without causing damage. 24 h later we injected 0.25 U neuraminidase to damage the ependyma [22] via an alternate mechanism. Histological examination of sections at 7 d revealed loss of the ependyma and the presence of RHO+ SVMs (Fig. 2d, left panels). Control injection of PBS vehicle alone resulted in no ependymal damage or SVM labelling (Fig. 2d, right panels). Thus, ependymal cell damage of diverse etiologies incites a reactive response by SVMs including phagocytosis of debris.

### Long-distance infiltration by SVMs after parenchymal injury

**Figure 3:**
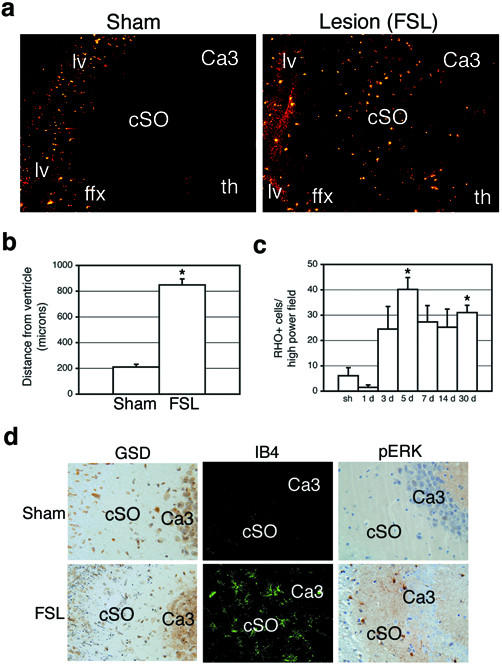
Infiltration of parenchyma by SVMs after injury. (A) SVMs infiltrated the *stratum oriens *of the hippocampus in injured mice (right panel) but not in sham animals (left panel). cSO, contralateral *stratum oriens *of hippocampus; ffx, fimbria/fornix; lv, lateral ventricle; th, thalamus. (B) SVMs migrate significantly farther into parenchyma of injured animals compared to sham injury (*p < 0.01). (C) Infiltration of hippocampus begins days after injury and cells remain for weeks (*p < 0.05 compared to sham). (D) Temporal pattern of infiltration corresponds to neuropil degeneration (black punctate staining, bottom left) activation of resident microglia (shown by increased IB4 staining, bottom middle) and glial activation (indicated by phospho-ERK immunoreactivity, bottom right). GSD, Gallyas silver degeneration stain; pERK, phospho-extracellular signal-related kinase.

Unpurturbed, SVMs remained in the immediate periventricular vicinity with no deeper parenchymal migration (Fig. 1c). Indeed, his population was stable in animals sacrificed up to 30d following dye injection (not shown). Periventricular lesions in HIVE and MS often extend deep into the parenchyma suggesting long-distance infiltration of reactive cells. Microglia have been shown to migrate *in vitro *in response to many chemokines and growth factors present in brain lesions and plaques [23-26]. In order to investigate whether activated SVMs can migrate towards parenchymal brain damage *in vivo *we gave mice a deafferenting lesion (FSL) of the hippocampus 24 hours following rhodamine dye injection and allowed survival for up to 28 days. Invasion of the parenchyma by RHO+ cells occurred in the *stratum oriens *of the denervated hippocampus (cSO) in 21/21 animals but not in sham animals (0/4) (Fig. 3a). Infiltrating cells were found an average of 849 ± 34 μm from the lateral ventricle after FSL compared to 210 ± 16 μm in uninjured mice (p < 0.01, Fig. 3b). Based on population distribution histograms, greater than 75% of RHO+ cells in sham animals were found within 300 μm of the ventricles (maximum: 860 μm) whereas greater than 75% were found beyond 400 μm (maximum: 2377 μm) in injured mice. Temporal quantification of RHO+ cell infiltration in the cSO demonstrates that this event commences between 1 and 3 days post-injury (PI) and peaks at 5 days PI (Fig. 3c). This timecourse mirrors that of the appearance of degeneration debris (Fig. 3d, GSD), activated resident hippocampal microglia (Fig. 3d, IB4), and reactive gliosis in the cSO (Fig. 3d, pERK [15]) supporting migration of RHO+ cells towards injury cues [24,25]. 94.6% of all RHO+ cells in the cSO were immunoreactive for F4/80 confirming the infiltrating cells are microglia. Finally, BrdU-positive/RHO+ cells were observed in the cSO maximally at the 3 day timepoint suggesting mitosis occurred primarily after the SVMs had migrated to the hippocampus (not shown). Therefore, activated SVMs are capable of infiltrating deep into the parenchyma in response to brain injury.

**Figure 4:**
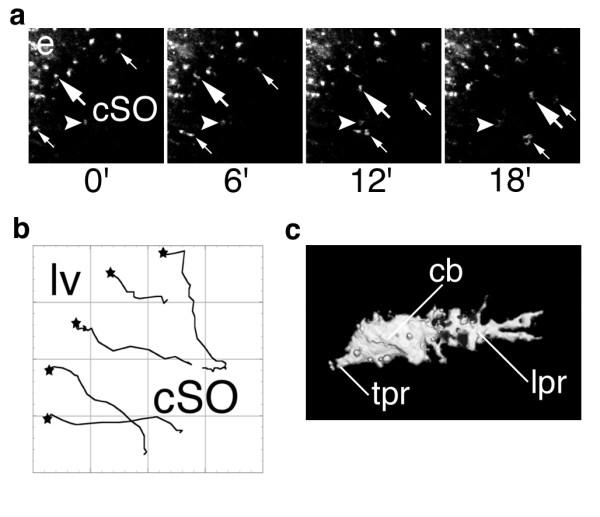
Dynamics of infiltrative microgliosis. (A) 2D projections of confocal images demonstrate three migratory cells (large and small white arrows) migrating into the cSO. white arrowhead, non-migratory cell for reference. e, ependyma; cSO, *stratum oriens*. See also Video 2. (B) Migration was highly directed from ventricle to hippocampus, five representative cells from a single experiment. lv, lateral ventricle; black stars, cell origin (C) Highly polarized, migratory morphologies of RHO+ cells as demonstrated by confocal 3D reconstruction. cb, cell body; lpr, leading process; tpr, trailing process.

To provide direct evidence for the migration of SVMs into the parenchyma and characterize their general migratory behavior we prepared live brain slices from dye-injected/lesioned mice and rendered confocal time-lapse movies in the cSO (Fig. 4a; Video 2 (Additional file 2)). RHO+ cells migrated in a directed fashion from the periventricular region into the cSO (Fig. 4b) and demonstrated an average speed of 80 ± 6 μm/h. Migrating cells displayed polarized morphologies with a prominent leading protrusion demonstrating numerous side branches (Fig. 4c). We conclude that activated SVMs are able to migrate long distances into the brain parenchyma towards damaged regions *in vivo *and *in situ*. We have named this event "infiltrative microgliosis" (IMG).

### ICV injection of HIV-1 tat protein causes IMG

**Figure 5:**
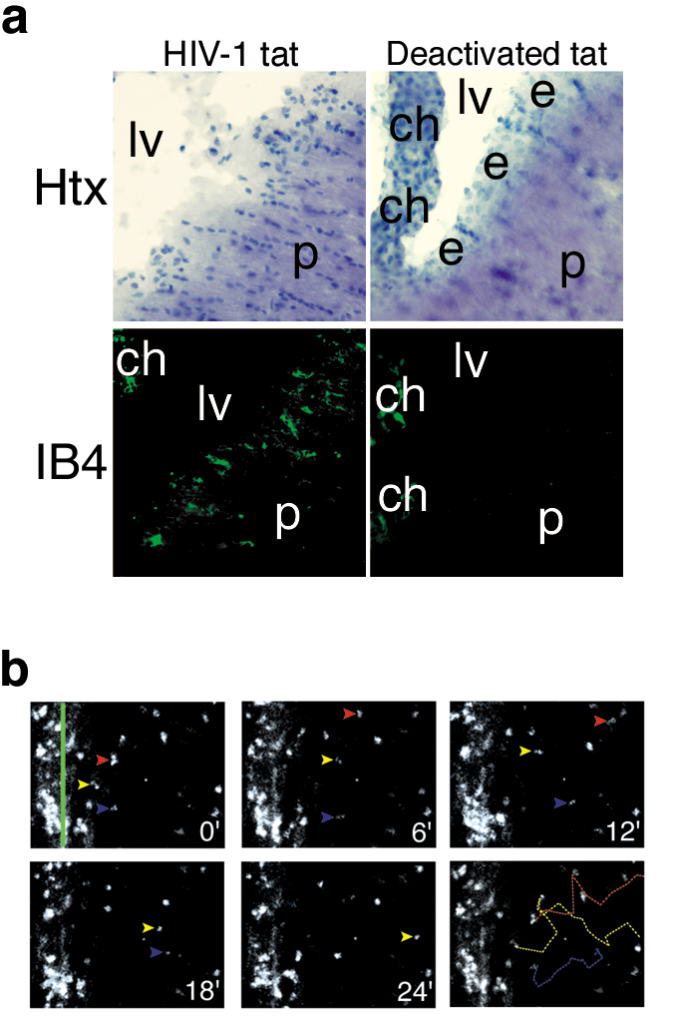
HIV-1 tat injection activates SVMs and incites IMG. (A) Ependymal loss (top left) and subventricular microgliosis (bottom left) 24 h following injection of 2.0 nmol tat protein but not in animals injected with deactivated tat (right panels). (B) To determine if tat-activated SVMs migrated *in situ *we rendered timelapse confocal movies 24 h post-injection. Colored arrowheads demonstrate three SVMs which migrate from the region near the ventricle (green line) deep into the parenchyma (colored dashed lines). Field measures ~200 μm horizontally. See also Video 5.

Lentiviral tat protein has been shown to be neurotoxic [8], stimulate microglial migration *in vitro *possibly by mimicking, and inducing expression of, chemokines [27,28], and soluble tat protein is released from HIV-infected cells [29]. Further, ependymal lesions were found in 16% of AIDS patients at autopsy [30] and HIV-1 tat has been shown to damage the ependymal layer of mice in low nanomolar concentrations [8]. To establish IMG as an event relevant to neurologic disease we tested the idea that ependymal damage caused by an ICV injection of 2.0 nM recombinant HIV-1 tat protein in mice would cause activation, and possibly intraparenchymal migration, of SVMs. 24 hours post-injection mice demonstrated ependymal cell damage (Fig. 5a, top left) and extensive activation of SVMs (Fig. 5a, bottom left). No damage or SVM activation was seen after injection of deactivated tat (Fig. 5a, right panels). Interestingly, activated microglia could often be found several hundred microns from ventricular surfaces as demonstrated by IB_4 _histochemistry (not shown). To determine whether this was due to migration of SVMs into the parenchyma or spreading activation of stationary cells we performed timelapse confocal analysis of live brain slices taken from animals 24 h after ICV tat injection. We found that nearly all activated periventricular microglia were motile and many were locomotory after tat injection (Video 3 (Additional file 3). Injection of deactivated tat did not result in migration (Video 4 (Additional file 4)). Velocities of HIV-1 tat activated microglia averaged approximately 500 μm/h. Further, we observed many microglia which migrated deep into the parenchyma from the periventricular zone (Fig. 5b, Video 5 (Additional file 5)). Intense microglial activity at the ependyma suggestive of phagocytosis was also observed (Video 5). We conclude that nanomolar concentrations of ICV-injected HIV-1 tat protein alone is sufficient to cause ependymal damage, SVM activation, and diffuse IMG.

## Discussion

We have shown that SVMs represent a pool of microglial cells which are highly reactive to periventricular damage and are responsible for clearance of resulting cellular debris. Further, activated SVMs are capable of migrating away from the ventricle towards injury cues from damaged regions in the parenchyma several hundred microns away. We confirmed these findings dynamically in acute slice preparations from adult mice. Both the juxtaventricular origin and the extensive migratory capacity of activated SVMs have important implications for neurobiology and disease.

Periventricular/subependymal microglia have been noted by histologists since microglia were first identified as a distinct cell type (reviewed in [31]). Little attention has been paid to these cells in the literature until recently due to their intimate arrangement among the subventricular neural progenitors [6,7]. Possible phenotypic differences between SVMs and parenchymal microglia have not been investigated, however, the location of SVMs among stem cells and within close proximity to ependymal cells and ventricular CSF is unique. A few neurological diseases demonstrate altered CSF constituents and often pathological involvement of the periventricular tissues [8-13]. Therefore, the specific function of microglia in these specialized regions of the brain under normal and pathological conditions deserve further investigation.

While evaluating rhodamine dyes for selective labeling of ependymal cells [21] for other studies we discovered that the ependyma of the injected hemisphere became rapidly damaged after dye uptake. Upon death of the ependyma, SVMs phagocytosed the ependymal debris, and thereby became rhodamine-positive. This serendipitous finding results in rapid and selective labelling of SVMs allowing study of this specific cell population *in vivo*. For instance, in this study we were able to demonstrate phagocytosis, mitosis, and migration of activated SVMs using histopathological techniques alone. We have shown these cellular activities can be confirmed *in situ *with timelapse confocal microscopy further validating this versatile protocol. Mechanistic investigations are possible by combining our *in vivo *and *in situ *protocols with genetic or pharmacological techniques.

We found SVMs only infiltrated the brain after selective ependymal damage with rhodamine dyes if a distant lesion was also present, likely providing a gradient of chemoattractive cues. CC chemokines are known to be upregulated rapidly after deafferenting injury of the hippocampus [26]. The extensive migration of SVMs in response to HIV-tat injection, on the other hand, may be due to a direct effect of tat on microglia or possibly an indirect effect due to upregulation of chemokines by neurons and glia [27,28]. Further, that ICV injection of recombinant HIV-1 tat protein *alone *is sufficient to damage the ependyma, activate SVMs, and incite infiltrative microgliosis supports the "cytokine dysregulation hypothesis" [8,22] of damage in HIV-1 encephalitis whereby overactivation of microglia/monocytes may be more critical than actual CNS viral load [33,34].

## Conclusions

In summary, we have shown that SVMs are a highly reactive pool of cells which, when activated, can infiltrate the parenchyma in response to injury cues from damaged brain regions or exposure to HIV-1 tat. These findings provide new *in vivo *and *in situ *models for the study of SVM function, further insight into microglial dynamics after brain injury, and novel hypotheses for the role of microglia in periventricular reactions in neurological diseases.

## List of abbreviations

BrdU, 5-bromo 2-deoxyuridine; BSA, bovine serum albumin; CSF, cerebrospinal fluid; cSO, *stratum oriens *of the hippocampus contralateral to stab lesion; DMSO, dimethyl sulfoxide; DTT, dithiothreitol; GFP, green fluorescent protein; GSD, modified Gallyas silver degeneration stain; HIVE, human immune deficiency virus 1 encephalitis; ICV, intracerebroventricular; IFN-γ, interferon gamma; IMG, infiltrative microgliosis; MS, multiple sclerosis; PBS, phosphate-buffered saline; pERK, phosphorylated/activated extracellular signal-regulated kinase; PI, post-injury; RHO+, rhodamine-positive; RhoB, rhodamine latex microbeads; SEM, standard error of the mean; Sp-DiI, 1,1'-dioctadecyl-6,6'-di(4-sulfophenyl)-3,3,3',3'-tetramethylindocarbocyanine; SVM, subventricular microglia; TNF-α, tumor necrosis factor alpha.

## Competing interests

The authors declare that they have no competing interests.

## Authors' contributions

WSC conceived of and designed the study, carried out all experiments, performed data analysis, and drafted the manuscript. S-IM participated in study design especially with regards to the timelapse experiments. AFH participated in study design and coordination and provided the confocal facilities, equipment, and expertise for timelapse experiments. JWM participated in study design and coordination and helped to draft the manuscript. All authors read and approved the final manuscript.

## Supplementary Material

Additional File 1 SVM phagocytosis of ependymal debris. Activity of SVMs suggestive of phagocytosis of dye-labeled ependymal cell debris 24 h following injection. SVMs can be seen extending processes towards debris. Examples of ependymal debris are pseudocolored yellow. Each frame is a 2D projection representing a stack of 6 images 8 μm apart. Original magnification, 40×. Click here for file

Additional File 2 Dynamics of infiltrative microgliosis. Infiltrative microgliosis of SVMs into the hippocampal *stratum oriens *from the subependymal region of the posterior lateral ventricle. Note highly directed migration into the hippocampus. Each frame is a 2D projection representing a stack of 4 images 10 μm apart taken every 3 minutes. Original magnification, 20×. Click here for file

Additional File 3 SVM dynamics in response to HIV-1 tat protein. Extensive migratory activation of periventricular microglia in response to 2.0 nM ICV HIV-1 tat protein. This migratory reaction extends several hundred microns into the parenchyma. v3v, ventral third ventricle. Each frame is a 2D projection representing a stack of 6 images 8 μm apart taken every 90 seconds. Original magnification, 20×. Field measures 700 × 700 μm. Click here for file

Additional File 4 Control video for HIV-1 tat protein. Lack of activation of SVMs and migration with ICV injection of deactivated HIV-1 tat protein (compare to Video 3, similar field). The paucity of IB4 labeling indicates the limited microglial activation. Note blood vessel endothelial cell labeling and gradual photobleaching. Each frame is a 2D projection representing a stack of 6 images 8 μm apart taken every 90 seconds. Original magnification, 20×. Field measures 700 × 700 μm. Click here for file

Additional File 5 HIV-1 tat incites infiltrative microgliosis. HIV-1 tat activated subventricular microglia infiltrate the parenchyma. Three highlighted cells correspond to those in Figure 5b. Note also intense activity of SVMs at ventricle (red line) suggestive of phagocytosis of ependymal cell debris. Each frame is a 2D projection representing a stack of 6 images 8 μm apart taken every 90 seconds. Original magnification, 40×. Click here for file
